# Prevalence and determinants of HealthHub app utilization among community-dwelling adults in Singapore

**DOI:** 10.1371/journal.pone.0327053

**Published:** 2025-07-17

**Authors:** Mahir Bakulkumar Goda, Alexander Shao-Rong Pang, Benedict Ding Chao Ong, Fang Nian Joanne Lim, Alvin Kian Wei Tan, Lay Hoon Goh

**Affiliations:** 1 Yong Loo Lin School of Medicine, National University of Singapore, Singapore, Singapore; 2 Ministry of Health Holdings Pte Ltd, Elementum, Singapore, Singapore; 3 Department of Family Medicine, National University Health System, Singapore, Singapore; 4 National University Polyclinics, Singapore, Singapore; Endeavour College of Natural Health, AUSTRALIA

## Abstract

**Introduction:**

Singapore’s aging population, projected to reach 18.7% by 2030, will increase chronic disease burden and strain healthcare resources. Digital health technologies, like Singapore’s HealthHub Application (HHA), are crucial for improving patient outcomes and healthcare delivery. However, the use of HHA among older adults in Singapore remains poorly understood. This study aimed to examine the prevalence of HHA use among community-dwelling Singaporeans and identify factors associated with its use.

**Methods:**

A cross-sectional survey was conducted in a public housing estate in Singapore, using random sampling to select housing units. Eligibility criteria included being a Singaporean or permanent resident, aged 40 years and above, possessing English proficiency, and absence of cognitive impairment. Participants completed a self-administered electronic questionnaire in English covering socio-demographics, health literacy, use of digital health technologies, and perceptions of HHA. Responses were categorized and analysed using descriptive statistics, univariate analysis, and multivariable logistic regression.

**Results:**

Of the 422 completed responses, 216 (51.2%) of participants reported HHA use. Higher HHA usage was associated with younger age, higher education levels, and greater self-perceived knowledge of health management. Older adults, particularly those aged 70 and above, and participants with secondary education or below had significantly lower HHA usage compared to their counterparts. Additionally, better health literacy as measured by positive perceptions of health knowledge were significantly associated with HHA usage.

**Conclusion:**

The adoption of digital health technologies like HHA is lower among older adults with lower education and health knowledge in Singapore. Targeted efforts to improve digital skills, awareness, and usability are needed to promote HHA uptake and support equitable healthcare access in an aging population.

## Introduction

The adoption of technology in healthcare offers a promising solution to reduce overall healthcare costs by improving the delivery of effective and efficient healthcare and supporting patients in their health management to optimise health outcomes [1–4]. This had proliferated in various forms, commonly utilized through digital health technologies (DHTs) such as mobile health, telehealth, and personalized medicine [5]. The COVID-19 pandemic had further accelerated the global uptake of DHTs, arising from initial efforts in the Organisation for Economic Co-operation and Development countries to scale up remote care to maintain healthcare access. In 2020, virtual consultations accounted for over 70% of primary care visits in Canada, 44% of appointments in Belgium, and one-third of visits in Costa Rica, driven by policy reforms, financial incentives, and changing patient preferences arising from the pandemic [6,7]. The successes of DHTs during this period showcased their promising potential and feasibility, thus significantly accelerated in its adoption in today’s current healthcare landscape.

In Singapore, the HealthHub application (HHA) is the national digital healthcare platform that provides health management services for all Singaporeans [8], providing one-stop access to their personal medical records, links to healthcare services (e.g., appointment booking), and preventive health promotion and tools (e.g., subsidized health programs) [9,10]. In 2023, two in three Singaporeans used HHA, totalling a million monthly users [11]. With Singapore’s population aged 65 years and older projected to rise from 8.5% in 2007 to 18.7% by 2030 [12], there will be an expected increase in chronic disease burden and healthcare demands [13]. The HHA is strategically positioned to address these challenges, playing a key role in empowering patients to manage their health more effectively while enhancing the efficiency and outcomes of healthcare delivery. However, majority of HHA users were younger Singaporeans, with only one in five adults above the age of 55 using the platform [14], highlighting a common challenge in the implementation and adoption of digital health technologies among older people [15,16].

Similar challenges have been observed internationally. In the UK, older adults aged 65 and above were almost 20 times less likely to have used a health application compared to those aged 18–24, despite over half expressing interest. Older adults were also significantly less likely to receive application recommendations from healthcare professionals, highlighting both user- and system-level barriers to digital health adoption [17]. Disparities in access, previous negative technological experiences, and insufficient technological support were common factors that hindered older adults’ uptake of digital health tools [18–20]. Conversely, key enablers for increasing use of digital health technologies in healthcare among older adults included higher education level, higher income, and better health literacy [21–27]. To date, there remains a paucity of literature on the use and key barriers and enablers for older Singaporeans to access, adopt, and use digital health technologies.

Hence, the objectives of this study were: (i) To examine the prevalence of HHA use among Singaporeans aged 40 and above, and (ii) identify factors associated with the use of HHA among Singaporeans aged 40 and above.

## Materials and methods

### Study design and recruitment

A cross-sectional survey-based study was performed from 12 to 14 December 2023 for residents living in public housing flats in Ang Mo Kio, a housing estate in the central part of Singapore. Using an estimated number of 2 million Singaporeans aged 40 and above as of June 2023, a 5% margin of error, and a 95% confidence level, a minimum sample size of 385 participants was required [28]. To ensure non-biased representation of the participants within Ang Mo Kio, we used random sampling to select the housing blocks for the study. When multiple eligible residents were present in a housing unit, the Kish Grid was used to select a resident for the study. To maximize participation and response rates, two follow-up visits were conducted within the week for household flats without any initial response. If there were still no response after two subsequent follow-up visits, the housing units were categorized as “non-responders”.

The inclusion criteria for the study were: (i) Singapore citizens or permanent residents, (ii) aged 40 years old and above, (iii) proficient in reading and understanding English, and (iv) not cognitively impaired. Participants who were not proficient in reading and understanding English were excluded from the study. To assess the presence of any cognitive impairment, the study team administered the Abbreviated Mental Test-4 [29]. Participants who obtained less than 4 points on the Abbreviated Mental Test-4 (i.e., answered at least one question wrongly) were considered to have abnormal cognition and were also excluded from the study.

Invitation letters describing the purpose and objectives of the study were distributed to the selected housing blocks within Ang Mo Kio. Participation in the study was voluntary with written informed consent obtained from the participants. The survey was self-administered door-to-door, and in English, with interviewer assistance if needed. Survey data was electronically collected and securely stored using Research Electronic Data Capture (REDCap), a web-based application developed to capture data for clinical research and create databases and projects [30].

### Survey and measurements

The study’s survey comprises 22 questions (S1 Appendix) and was developed using constructs from the Technology Acceptance Model (TAM) [31] and items adapted from validated instruments used in prior studies investigating technology acceptance in various healthcare settings, including assessing participants’ views on their health, health literacy, use of DHTs, and perceptions of ease of use and usefulness of digital platforms [32,33].

The 22 questions were divided into three main sections. The first section collected socio-demographic data including age, gender, ethnicity, educational level, and average household income. The second section assessed the participants’ use of digital health technologies and health literacy based on key dimensions of Sørensen et al.’s integrated model of health literacy [34], specifically participants’ self-reported understanding of their health conditions, confidence in managing their health, and perceived knowledge of health management. This section also included questions on mobile technology use for health-related purposes and access to regular healthcare services. The third section consisted of (i) a standardized set of infographics and instructions describing HHA’s interface, key features, and functionality to standardize the participants’ understanding of how HHA could be used, and (ii) questions evaluating the HHA based on the information given to them, across four domains of clarity, ease of use, relevance, and utility.

When appropriate, questions were presented as statements and responses were recorded on a 7-point scale. Responses were later re-categorized into three groups: (i) “Strongly Disagree”, “Disagree”, and “Somewhat Disagree” were grouped together as “Disagree”, (ii) “Strongly Agree”, “Agree”, and “Somewhat Agree” were grouped together as “Agree”, and (iii) “Neutral” remained as “Neutral”. Factual and categorical items, such as those related to HHA usage and participant demographics, were presented in alternative formats as appropriate.

Content and face validation were conducted with nine conveniently sampled Singapore residents aged 40 and above to ensure clarity, relevance, and contextual appropriateness for local users. These forms of validation were selected to ensure comprehensibility for the target population during early tool development. Based on their feedback, amendments were made to contextualize the survey items to the HHA [31–33]. Construct and criterion validity were not conducted, as the survey was intended for exploratory, cross-sectional use rather than the development of a generalizable instrument.

In this study, HHA use was defined as having reported prior or current use of HHA at the point of administering the survey.

### Statistical analysis

Descriptive analysis using frequencies and percentages was performed to examine the demographic data, health literacy, use of digital health technologies factors, and HHA usage. A univariate analysis was performed to identify variables associated with HHA usage, using simple binary logistic regression. Multivariable logistic regression with forward likelihood ratio selection was then used to examine the covariate effects of each variable on HHA usage. Variables with a univariate *p*-value of <0.2 were included in the multivariable logistic regression [35]. Odds ratio (OR) and 95% confidence interval (CI) were reported for both univariate and multivariate analyses. GraphPad Prism Version 10.1.2 was used for statistical analyses with a significance threshold of *p*-value < 0.05 [36]. There was no missing data.

### Ethics approval

Ethics approval for the study was obtained from the National University of Singapore Saw Swee Hock School of Public Health Departmental Ethics Review Committee (Ref: SSHSPH-204). The study involved anonymised surveys without the collection of personal identifiers; information collected were not directly linked to the participants. The study was conducted in compliance with the University’s institutional policies, regulations, and guidelines.

## Results

### Characteristics of participants

A total of 454 eligible individuals participated in the study, of whom 422 participants completed the survey, yielding a response rate of 93.0%. The final sample comprised a diverse cohort of Singaporeans aged 40 years and above. Among them, 143 (33.9%) were aged 70 years and above, 104 (24.6%) were aged 60–69, 91 (21.6%) were aged 50–59, and 84 (19.9%) were aged 40–49 (Table 1). Of the 422 participants, 227 (53.8%) were male. In terms of ethnicity, 321 (76.1%) were Chinese, followed by 45 (10.7%) were Indian, 45 (10.7%) were Malay, and 11 (2.6%) were of other ethnicities. In terms of education and income, 249 (59.0%) had attained the highest education level of secondary school and below, and 98 (23.2%) had an average household income of less than SGD2,000.

**Table 1:** Characteristics of participants in study.

Total no. of participants	Used HHA before	Has not used HHA before
**(n = 422)**	**(n = 216)**	**(n = 206)**
***Age Group (years)***		
40-49	67 (31.0%)	17 (8.3%)
50-59	60 (27.8%)	31 (15.1%)
60-69	51 (23.6%)	53 (25.7%)
≥ 70	38 (17.6%)	105 (51.0%)
***Gender***		
Female	98 (45.4%)	97 (47.1%)
Male	118 (54.6%)	109 (52.9%)
***Ethnicity***		
Chinese	156 (72.2%)	165 (80.1%)
Indian	28 (13.0%)	17 (8.3%)
Malay	25 (11.6%)	20 (9.7%)
Others	7 (3.2%)	4 (1.9%)
***Highest Education Level Attained***		
Secondary^1^ & below^2^	82 (38.0%)	167 (81.1%)
Post-Secondary Education^3^ & Diploma^4^	74 (34.3%)	23 (11.2%)
Degree^5^	60 (27.8%)	16 (7.8%)
***Average Household Income (SGD)***		
< 2,000	33 (15.3%)	65 (31.6%)
2,000-3,999	26 (12.0%)	25 (12.1%)
4,000-5,999	37 (17.1%)	14 (6.8%)
6,000-9,999	22 (10.2%)	7 (3.4%)
≥ 10,000	25 (11.6%)	5 (2.4%)
Prefer not to say	73 (33.8%)	90 (43.7%)

*Note:* HHA = HealthHub application.

^1^At least 1 GCE ‘N’/’O’ Level pass, National Institute of Technical Education (ITE) Certificate (Intermediate), ITE Skills Certificate (ISC), or at least 3 ESS WPLN Statements of Attainment at Level 5 and above.

^2^No Qualification, Primary Education or Lower Secondary Education.

^3^At least 1 GCE Advanced (‘A’)/Higher 2 (‘H2’) Level pass, National ITE Certificate (Nitec)/Higher Nitec/Master Nitec, Workforce Skills Qualifications (WSQ) Certificate/Higher Certificate/Advanced Certificate, International Baccalaureate/High school diploma, or other certificates/qualifications of equivalent standard.

^4^Polytechnic Diplomas, Advanced Diplomas or Post-Diploma Certificates.

^5^Bachelor’s Degree, Postgraduate Diploma, Master’s Degree or Doctorate.

Among the 422 participants, 216 (51.2%) reported current or prior use of HHA (Table 1). Among HHA users, 127 (58.8%) were 40–59 years of age, 118 (54.6%) were male, 156 (72.2%) were Chinese, and 134 (62.1%) had attained the highest education level of post-secondary school and above. The average household incomes of HHA users were distributed across the categories: 33 users (15.3%) reported earning less than SGD 2,000, 26 (12.0%) earned between SGD 2,000–3,999, 37 (17.1%) earned between SGD 4,000–5,999, 22 (10.2%) earned between SGD 6,000–9,999, and 25 (11.6%) earned SGD 10,000 or more. The remaining 73 (33.8%) preferred not to disclose their income. Among the 206 (48.8%) non-HHA users, 158 (76.7%) were 60 years and older, and 109 (52.9%) were male. Compared to HHA users, a greater proportion had lower educational attainment with 167 (81.1%) reporting the highest education level of secondary school or below. Additionally, 65 (31.6%) of non-HHA users reported household income of less than SGD 2,000. 

### Participants’ health literacy and use of digital health technologies

Table 2 presents participants’ self-reported health literacy, healthcare access, and attitudes toward mobile devices, stratified by HHA usage. Across both HHA users and non-users, the majority of participants agreed with statements reflecting good perceived health status and high self-reported health literacy. Notably, 164 (75.9%) HHA users and 144 (69.9%) non-users agreed that they were in good health. Similarly, 182 (84.3%) of HHA users and 146 (70.9%) of non-users agreed that they were knowledgeable about managing their health conditions or problems. This trend continued with their understanding of their health conditions, with 186 (86.1%) of HHA users and 155 (75.2%) of non-users agreeing. In terms of healthcare access, 157 (72.7%) of HHA users and 148 (71.8%) of non-users reported having a regular healthcare provider.

**Table 2:** Participants’ health literacy and use of digital health technologies.

Factor	Use of HHA	Does not use HHA
	**(n = 216)**	**(n = 206)**
***I am in good health***		
Disagree	29 (13.4%)	42 (20.4%)
Neutral	23 (10.6%)	20 (9.7%)
Agree	164 (75.9%)	144 (69.9%)
***I am very knowledgeable regarding care for my health conditions or problems***		
Disagree	9 (4.2%)	28 (13.6%)
Neutral	25 (11.6%)	32 (15.5%)
Agree	182 (84.3%)	146 (70.9%)
***I understand my health conditions or problems and how to care for them***		
Disagree	8 (3.7%)	21 (10.2%)
Neutral	22 (10.2%)	30 (14.6%)
Agree	186 (86.1%)	155 (75.2%)
***I have a regular doctor at a GP clinic, polyclinic, or hospital whom I visit for my healthcare needs***		
Disagree	48 (22.2%)	49 (23.8%)
Neutral	11 (5.1%)	9 (4.4%)
Agree	157 (72.7%)	148 (71.8%)
***I have a chronic disease that requires me to see a doctor regularly***		
Disagree	107 (49.5%)	84 (40.8%)
Neutral	12 (5.6%)	9 (4.4%)
Agree	97 (44.9%)	113 (54.9%)
Having access to mobile device(s)* is important to me		
Disagree	10 (4.6%)	59 (28.6%)
Neutral	11 (5.1%)	13 (6.3%)
Agree	195 (90.3%)	134 (65.0%)
The ability to send and receive information (e.g., email, messaging platforms, social media, Instagram, apps) via mobile device(s)* is important to me		
Disagree	13 (6.0%)	71 (34.5%)
Neutral	14 (6.5%)	23 (11.2%)
Agree	189 (87.5%)	112 (54.4%)

*Note:* HHA = HealthHub application; *Mobile Device: laptop, tablet, iPad, smartphone.

Regarding the presence of chronic disease, 107 HHA users (49.5%), disagreed that they had a condition requiring regular medical attention, compared to 84 (40.8%) non-HHA users.

More pronounced differences were observed in attitudes toward mobile devices. Among HHA users, 195 (90.3%) agreed that access to mobile devices was important to them, compared to 134 (65.0%) of non-users. Similarly, 189 (87.5%) of HHA users valued the ability to send and receive information via mobile devices, in contrast to 112 (54.4%) of non-users. 

### Participants’ perceived ease of use and usefulness of HHA

Table 3 shows insights on participants’ perceived ease of use and usefulness of HHA in four key categories: clarity, ease of use, relevance, and utility. For clarity, 261 (61.8% of 422) participants found HHA clear and understandable, compared to 90 (21.3%) of participants who did not. For ease of use, 251 (59.5%) participants found HHA easy to use, compared to 106 (25.1%) of participants who did not. Additionally, 242 (57.3%) participants found it easy to accomplish their goals using HHA, in contrast to 109 (25.8%) participants who perceived otherwise. For relevance, 259 (61.4%) participants agreed that HHA supports important aspects of their healthcare, in contrast to 109 (25.8%) participants who disagreed. For utility, 250 (59.2%) participants recognised HHA’s utility in improving healthcare, compared to 115 (27.3%) participants who believed otherwise. Furthermore, 251 (59.5%) participants agreed that HHA was useful in managing healthcare, while 111 (26.3%) participants did not.

**Table 3:** Participants’ perceived ease of use and usefulness of HHA.

Category/Item	Disagree	Neutral	Agree
***Clarity***			
HHA was clear and understandable	90 (21.3%)	71 (16.8%)	261 (61.8%)
***Ease of Use***			
HHA was easy to use	106 (25.1%)	65 (15.4%)	251 (59.5%)
Easy to get the App to do what I want it to do	109 (25.8%)	71 (16.8%)	242 (57.3%)
***Relevance***			
HHA supported important aspects of the user’s healthcare	109 (25.8%)	54 (12.8%)	259 (61.4%)
***Utility***			
Using the HHA increases user’s effectiveness in managing my healthcare	115 (27.3%)	57 (13.5%)	250 (59.2%)
HHA was useful in managing the user’s healthcare	111 (26.3%)	60 (14.2%)	251 (59.5%)

*Note:* HHA = HealthHub application.

### Univariate analysis of variables associated with HHA usage

Based on the univariate analysis, age, highest education level attained, and average household income were statistically significant with HHA usage (Table 4). In addition, agreement with the statements about having knowledge and understanding regarding care for health conditions or problems, importance of access to mobile devices, and importance of ability to send and receive information via mobile devices were also statistically significant with HHA usage. 

**Table 4:** Univariate analysis of factors associated with HHA usage.

Variable	Odds Ratio (95% CI)	*p*-value
***Age Group (years)***		<0.001
40–49 (reference)	1	
50-59	0.49 (0.24-0.97)	0.042
60-69	0.24 (0.12-0.46)	<0.001
≥ 70	0.09 (0.05-0.17)	<0.001
***Gender***		0.760
Female (reference)	1	
Male	1.07 (0.73-1.57)	0.724
***Ethnicity***		0.141
Chinese (reference)	1	
Indian	1.74 (0.93-3.37)	0.090
Malay	1.32 (0.71-2.50)	0.383
Others	1.85 (0.55-7.18)	0.334
***Highest Education Level Attained***		<0.001
Secondary^1^ & below^2^ (reference)	1	
Post-Secondary Education^3^ & Diploma^4^	6.55 (3.88-11.41)	<0.001
Degree^5^	7.64 (4.23-14.48)	<0.001
***Average Household Income (SGD)***		<0.001
< 2,000 (reference)	1	
2,000-3,999	2.05 (1.03-4.11)	0.042
4,000-5,999	5.21 (2.52-11.25)	<0.001
6,000-9,999	6.19 (2.50-17.04)	<0.001
≥ 10,000	9.85 (3.71-31.31)	<0.001
Prefer not to say	1.60 (0.95-2.71)	0.078
***I am in good health***		0.211
Neutral (reference)	1	
Agree	0.99 (0.52-1.88)	0.976
Disagree	0.60 (0.28-1.29)	0.190
***I am very knowledgeable regarding care for my health conditions or problems***		0.011
Neutral (reference)	1	
Agree	1.60 (0.91-2.83)	0.106
Disagree	0.41 (0.16-1.00)	0.057
***I understand my health conditions or problems and how to care for them***		0.044
Neutral (reference)	1	
Agree	1.64 (0.91-2.98)	0.102
Disagree	0.52 (0.19-1.36)	0.192
***I have a regular doctor at a GP clinic, polyclinic, or hospital whom I visit for my healthcare needs***		0.716
Neutral (reference)	1	
Agree	0.87 (0.34-2.16)	0.760
Disagree	0.80 (0.30-2.11)	0.654
***I have a chronic disease that requires me to see a doctor regularly***		0.074
Neutral (reference)	1	
Agree	0.64 (0.25-1.59)	0.341
Disagree	0.96 (0.37-2.36)	0.922
Having access to mobile device(s)* is important to me		<0.001
Neutral (reference)	1	
Agree	1.72 (0.75-4.03)	0.202
Disagree	0.20 (0.07-0.57)	0.003
The ability to send and receive information (e.g., email, messaging platforms, social media, Instagram, apps) via mobile device(s)* is important to me		<0.001
Neutral (reference)	1	
Agree	2.77 (1.37-5.73)	<0.005
Disagree	0.30 (0.12-0.73)	<0.008

*Note:* HHA = HealthHub application, 95% CI = 95% confidence interval.

^1^At least 1 GCE ‘N’/’O’ Level pass, National ITE Certificate (Intermediate), ITE Skills Certificate (ISC), or at least 3 ESS WPLN Statements of Attainment at Level 5 and above.

^2^No Qualification, Primary Education or Lower Secondary Education.

^3^At least 1 GCE Advanced (‘A’)/Higher 2 (‘H2’) Level pass, Nitec/Higher Nitec/Master Nitec, Workforce Skills Qualifications (WSQ) Certificate/Higher Certificate/Advanced Certificate, International Baccalaureate/High school diploma, or other certificates/qualifications of equivalent standard.

^4^Polytechnic Diplomas, Advanced Diplomas or Post-Diploma Certificates.

^5^Bachelor’s Degree, Postgraduate Diploma, Master’s Degree or Doctorate.

*Mobile Device: laptop, tablet, iPad, smartphone.

Gender and ethnicity were not statistically significant with HHA usage. Responses to the statements on health status, having a regular doctor for healthcare needs, and having chronic conditions that require seeing a doctor regularly, were also not statistically significant with HHA usage. 

### Multivariate analysis of variables associated with HHA usage

Multivariable logistic regression was used to examine the covariate effects of each variable on HHA usage (Table 5). The regression model included all variables that showed statistical significance in the univariate analysis. In this model, statistically significant variables associated with use of HHA included attaining the highest education level of Post-Secondary Education and Diploma (OR 3.36, 95% CI [1.85–6.22]) and Degree (OR 3.19, 95% CI [1.62–6.51]) when compared against Secondary or below. Additionally, agreement with the statement of being knowledgeable regarding care for their health conditions or problems (OR 2.41, 95% CI [1.20–4.95]) was also statistically significant with HHA usage, when compared against those that were neutral to the statement. In the same model, statistically significant variables for not using HHA include older age groups of 60–69 years old (OR 0.44, 95% CI [0.20–0.92]) and 70 years old and older (OR 0.27, 95% CI [0.12–0.58]) when compared against a reference age group of 40–49 years old. 

**Table 5:** Multivariable logistic regression for variables associated with HHA usage.

Variable	Odds Ratio (95% CI)	*p*-value
***Age Group (years)***		
40 - 49 (reference)	1	
60 - 69	0.44 (0.20-0.92)	0.031
≥70	0.27 (0.12-0.58)	0.001
***Highest Education Level Attained***		
Secondary^1^ & below^2^ (reference)	1	
Post-Secondary Education^3^ & Diploma^4^	3.36 (1.85-6.22)	< 0.001
Degree^5^	3.19 (1.62-6.51)	0.001
***I am very knowledgeable regarding care for my health conditions or problems***		
Neutral (reference)	1	
Agree	2.41 (1.20-4.95)	0.015

*Note:* HHA = HealthHub application, 95% CI = 95% confidence interval.

Model adjusted to age, Highest Education Level Attained, responses to the statements regarding Knowledgeable regarding Care for Health Conditions or Problems, Importance of Access to Mobile Device(s), Importance of Ability to Send, and Receive Information via Mobile Device(s).

^1^At least 1 GCE ‘N’/’O’ Level pass, National ITE Certificate (Intermediate), ITE Skills Certificate (ISC), or at least 3 ESS WPLN Statements of Attainment at Level 5 and above.

^2^No Qualification, Primary Education or Lower Secondary Education.

^3^At least 1 GCE Advanced (‘A’)/Higher 2 (‘H2’) Level pass, Nitec/Higher Nitec/Master Nitec, Workforce Skills Qualifications (WSQ) Certificate/Higher Certificate/Advanced Certificate, International Baccalaureate/High school diploma, or other certificates/qualifications of equivalent standard.

^4^Polytechnic Diplomas, Advanced Diplomas or Post-Diploma Certificates.

^5^Bachelor’s Degree, Postgraduate Diploma, Master’s Degree or Doctorate.

## Discussion

In this study, the prevalence of HHA use was found to be 51.2% among community-dwelling adults in Singapore. The study discovered that the key factors associated with increased HHA usage were higher educational status, perception of being knowledgeable regarding care for their health conditions, and younger age groups of 40–49 years old. Although this prevalence is higher than reported in an early study [14], the overall uptake of HHA leaves much to be desired.

A key finding was the significant association between older age and not using HHA use. This is consistent with existing literature that showed lower use of digital health technologies and reduced digital device ownership among older adults [37]. Unwillingness to use health applications was also associated with older age due to limited familiarity with navigating mobile platforms [17]. It is important to invest efforts to identify and address potential barriers faced by older people, thus empowering them to be more actively engaged in managing their health by using digital health platforms. With chronic diseases occurring more frequently with increasing age in Singapore, older adults are likely to reap the benefits from the use of HHA.

The study also found that the highest level of education attained influenced the use of HHA. Participants with higher educational levels were more likely to use HHA, which remained statistically significant even when adjusted for age, perceptions of health knowledge, and attitudes towards mobile devices. Consistent with existing literature, Soundararajan et al. identified having lower education levels as a potential barrier to the uptake of digital health skills [38].

Another key finding of this study was that individuals with higher health literacy were more likely to use HHA. Specifically, participants who reported stronger self-perceived knowledge of health management were more likely to use HHA. This finding aligns with evidence that active health management correlates with higher patient activation, defined as the ability to take an active role in one’s healthcare [39,40]. Thus, individuals with health-seeking behaviours are more inclined to use tools such as HHA to support their healthcare. A bidirectional relationship may also exist, whereby HHA use increases health knowledge and confidence in managing one’s health.

To improve HHA adoption in the population, efforts should target older adults, people with lower educational levels, and who are less knowledgeable about health management. Collaborations with community partners serving the elderly and lower socioeconomic groups could help overcome these specific barriers. These community partners provide digital health education workshops and educational campaigns, such as the “Seniors Go Digital” and “Seniors for Smart Nation” programs [41], and one-to-one coaching for senior citizens on the general use of smartphones and devices.

While most participants in our study reported positive impressions of HHA regarding clarity, ease of use, relevance, and utility, which was consistent with existing evidence [42], there is room for improvement in the design and functionality of HHA. Gomez-Hernandez et al. recommend enlarging and spacing out interactive controls for better usability [43], while Fischer et al. suggest simplifying interfaces to enhance ease of use for older adults [44]. Additional enhancements could include gamification that integrates game-like elements to create engaging and rewarding user experiences, which has shown positive effects in motivating rehabilitation and health management for people with cardiovascular diseases [45–47]. Personalization features, such as tailored advice for managing specific conditions and medication reminders, have also been shown to be beneficial [48]. Furthermore, the use of conversational agents to promote healthy lifestyle changes has been well received, as demonstrated in a 2021 local study by Dhinagaran et al [49].

Although design improvements are not the primary barrier to HHA adoption in our study, enhancements could significantly complement efforts to improve use of digital health technologies and familiarity with digital tools in older adults [50,51]. Design improvements could support greater adoption and sustained use across all age groups [52]. Future studies should explore these design modifications to ensure the application is as user-friendly and inclusive as possible.

Although HHA is unique to Singapore, the findings from this study offer transferable insights for other national or institutional DHTs. The identified barriers and enablers, particularly around age, education, and perceived health knowledge, mirror trends observed internationally, suggesting that strategies such as tailored onboarding support for specific population groups and simplified interfaces may be universally applicable. These results may also inform the iterative development of similar tools in urbanized, ageing societies where digital engagement is uneven.

Despite the increasing importance of digital health services for accessing public healthcare, older individuals remain underrepresented in their usage, posing a challenge to the principles of equal access and care delivery based on need [53]. Further research is needed to explore these issues, particularly by identifying the barriers faced by non-English-speaking and less digitally proficient populations. Longitudinal studies are needed to assess whether initial uptake of HHA translates into sustained use, improved health behaviours, and long-term outcomes to demonstrate the real-world benefits of these digital technologies.

## Limitations

First, given that this was a cross-sectional study, causal relationships or the direction of these associations cannot be established. However, information on the prevalence of the use of HHA, and highlighted factors that are enablers or barriers have been provided, which can be further investigated in long-term studies.

Second, the use of self-reported, closed-ended questionnaires might have introduced recall and perception biases. In addition, questions regarding participants’ health literacy and use of digital health technologies were also subjective, and responses were solely based on their interpretations of the questions. There could also have been acquiescence bias as surveys were conducted face-to-face so participants might have been inclined to give more positive responses to the survey statements without accurately reflecting their own views.

Third, given that the HHA was only available in the English language, the study assumes that HHA users were proficient in English and hence participants not proficient in English were excluded. Along with the use of English-only questionnaires and illustrations of HHA, selection bias had been introduced into the sampling process. Furthermore, no formal language assessment was conducted beyond the participants’ self-reporting of their ability to understand the study process, questionnaires, and illustrations. By excluding non-English proficient individuals, the study results were skewed towards English-proficient participants who might be younger, be from a higher socio-economic status, and better educated, and thus would not be fully representative of Singapore’s older population. Future research should use translated questionnaires and include participants across all major language groups to obtain a more comprehensive understanding of the community’s use and perception of HHA.

Fourth, factors associated with HHA usage found in this study may not be fully generalizable to other digital health applications, especially if they serve different functions from HHA. For example, during the COVID-19 pandemic, TraceTogether and SafeEntry were digital health applications with a legal mandate to be adopted by all Singaporeans for the purpose of contact tracing [54,55]. This had led to widespread use and normalcy of these applications without similar implementation challenges seen with HHA as reflected in this study.

Finally, our sample may not be fully representative, with possible under-representation of individuals from lower socioeconomic backgrounds, minority ethnic groups, and those with limited health or digital literacy. Future research should adopt more inclusive sampling strategies to ensure adequate representation across socioeconomic, ethnic, and health literacy groups, thereby enhancing generalizability and understanding of adoption barriers across diverse populations.

## Conclusion

This study highlights key factors influencing the use of HHA in Singapore, particularly among older adults and those with varying educational levels and knowledge of health management. Despite a relatively higher prevalence of HHA use in our sample than in previous studies, the overall adoption remains lacking, especially among older and less digitally literate populations. Addressing barriers such as limited digital skills, lack of awareness of digital health technologies, and usability challenges can promote greater HHA adoption. Strengthening efforts to improve use of digital health technologies, expanding targeted information campaigns, and refining application design could further ensure that more individuals can benefit from the advantages of digital health platforms. By doing so, this approach supports a move toward a more equitable and effective healthcare delivery in the digital age.

## Supporting information

S1 Appendix Survey Questionnaire. (DOCX)
