# Supplementary material for: Prevalence and determinants of HealthHub app utilization among community-dwelling adults in Singapore
Source: PLoS One. 2025 Jul 17;20(7):e0327053. doi: 10.1371/journal.pone.0327053 (PMC12270099; doi:10.1371/journal.pone.0327053)
Supplement: S1 Appendix — (DOCX) [file pone.0327053.s001.docx]

**S1 Appendix – Survey Questionnaire**

**Title:** Prevalence and Determinants of HealthHub App Utilization Among Community-Dwelling Adults in Singapore

**Section A: Socio-demographics**

This section contains **8** questions on your personal details.

1. Nationality:

- Singapore Citizen
- Singapore Permanent Resident (PR)

1. Age (on date of survey): ______ years old

1. Gender:

- Male
- Female

1. Ethnicity:

- Chinese
- Malay
- Indian
- Others (Please specify: __________________________)

1. What is your highest level of education?

- No formal education
- Primary School
- Secondary School
- Post-Secondary Education (Junior College/ITE)
- Polytechnic/Private diploma
- University and above

1. Which of the following best describes your main work status* over the last 12 months?

** Refers to what you spent most of the usual working hours on during the last 12 months.*

- Student
- Working
- Homemaker or Housewife
- Retired
- Unemployed

1. Over the last 12 months, what is the **total average household income (S$) in a month**, before any deductions?

- Below $2,000 per month
- $2,000 - 3,999 per month
- $4,000 - 5,999 per month
- $6,000 - 9,999 per month
- $10,000 - 14,999 per month
- $15,000 and above per month
- Prefer not to say

1. Excluding yourself, how many other people live in your household? Include domestic helper and tenants/relatives.

- None, I live alone
- 1
- 2
- 3
- 4
- 5
- More than 5 (Please specify the number: _______)

**Section B: Questions about Health**

This section contains **7** questions to help us find out more about your health. There are no correct or wrong answers, so please answer to the best of your ability.

1. How much would you agree or disagree with this statement: **“I am in good health”**?

| Strongly Disagree | Disagree | Somewhat Disagree | Neutral | Somewhat Agree | Agree | Strongly Agree |
| --- | --- | --- | --- | --- | --- | --- |

1. I am very knowledgeable regarding care for my health conditions or problems.

| Strongly Disagree | Disagree | Somewhat Disagree | Neutral | Somewhat Agree | Agree | Strongly Agree |
| --- | --- | --- | --- | --- | --- | --- |

1. I understand my health conditions or problems and how to care for them.

| Strongly Disagree | Disagree | Somewhat Disagree | Neutral | Somewhat Agree | Agree | Strongly Agree |
| --- | --- | --- | --- | --- | --- | --- |

1. Having access to mobile device(s)* is important to me.

**Mobile device(s): laptop, tablet, iPad, smartphone*

| Strongly Disagree | Disagree | Somewhat Disagree | Neutral | Somewhat Agree | Agree | Strongly Agree |
| --- | --- | --- | --- | --- | --- | --- |

1. The ability to send and receive information (e.g., email, messaging platforms, social media, Instagram, apps) via mobile device(s)* is important to me.

**Mobile device(s): laptop, tablet, iPad, smartphone*

| Strongly Disagree | Disagree | Somewhat Disagree | Neutral | Somewhat Agree | Agree | Strongly Agree |
| --- | --- | --- | --- | --- | --- | --- |

1. I have a regular doctor at a GP clinic, polyclinic, or hospital whom I visit for my healthcare needs.

| Strongly Disagree | Disagree | Somewhat Disagree | Neutral | Somewhat Agree | Agree | Strongly Agree |
| --- | --- | --- | --- | --- | --- | --- |

1. I have a chronic disease (such as diabetes or asthma) that requires me to see a doctor regularly.

| Strongly Disagree | Disagree | Somewhat Disagree | Neutral | Somewhat Agree | Agree | Strongly Agree |
| --- | --- | --- | --- | --- | --- | --- |

# **Section C: Explainer on HealthHub App**

This Section C is for you to read and understand how to access and use HealthHub app before answering the questions in Section D. You are not required or expected to sign in or sign up for HealthHub app.

1. Log-in page


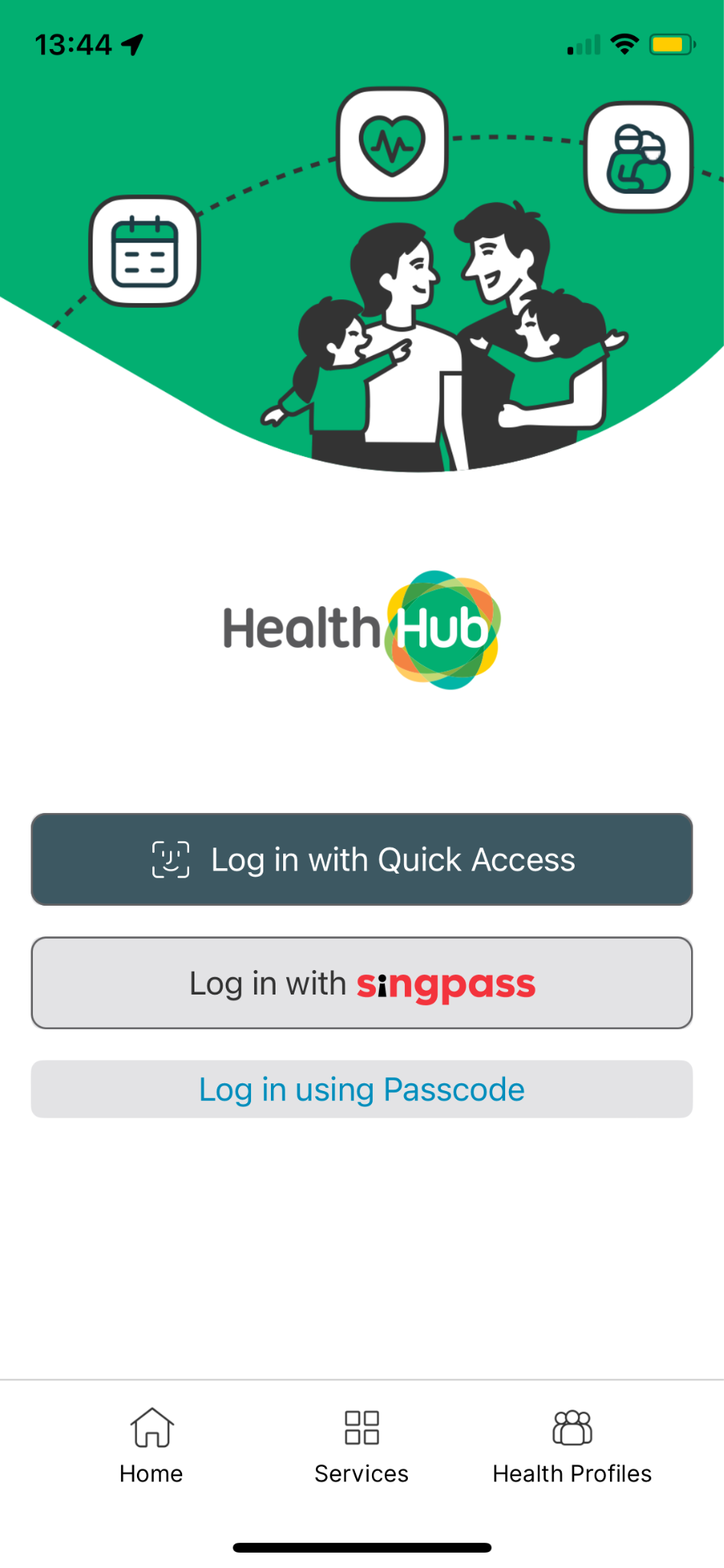

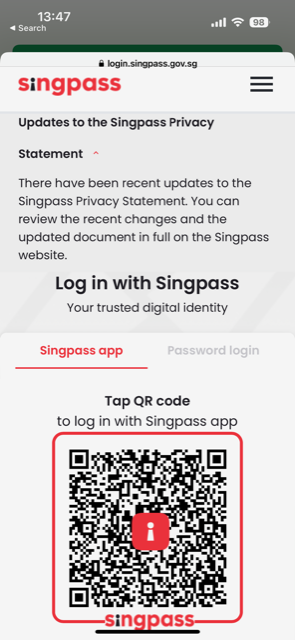


Screenshot 1: Log-in page of the HealthHub application

You can use the **Log-In Page** to log into your Health Hub Application using your Singpass Account upon your first time using the application. After this, you can set-up 2 other log in methods: using your phone’s Face Recognition or Fingerprint Security Function or using a Passcode set by you.

1. Application home page


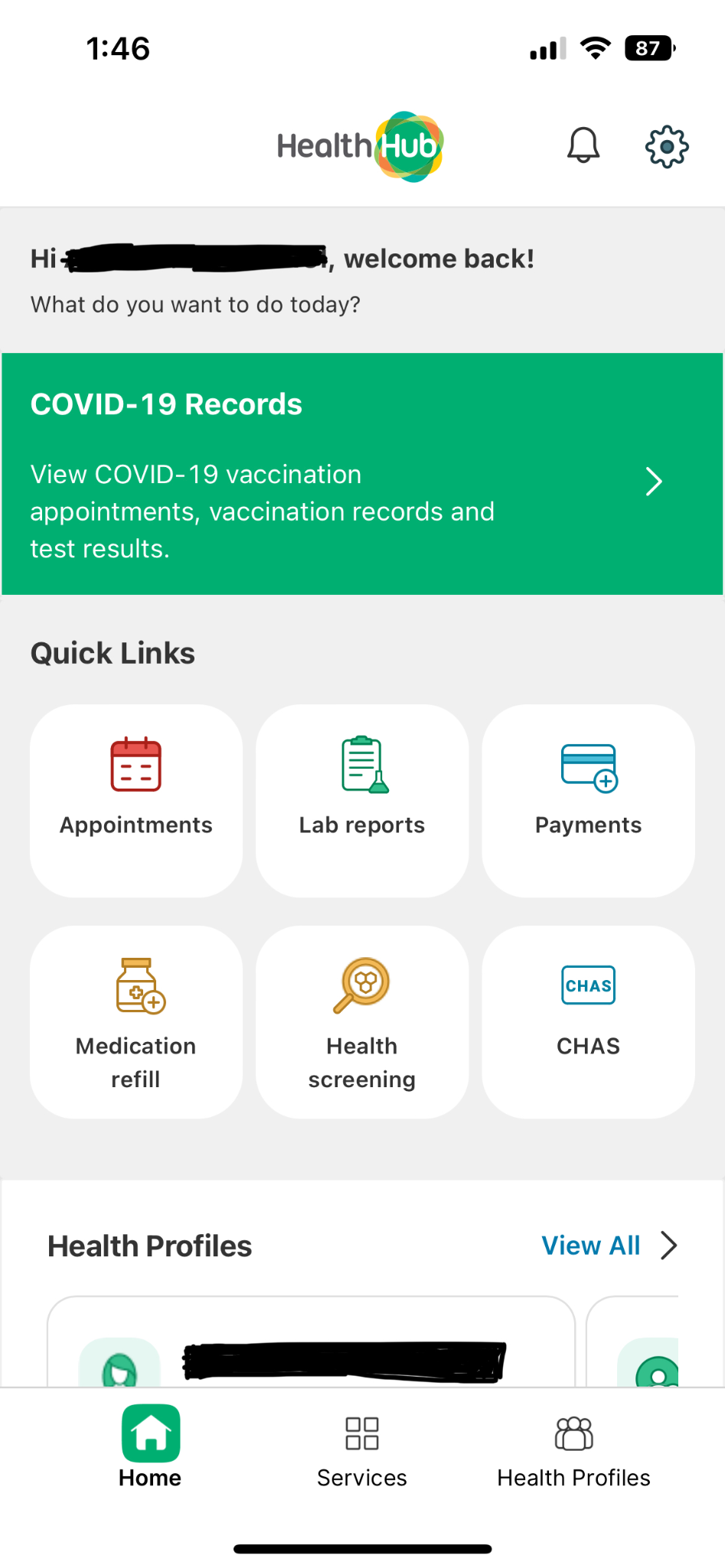


Screenshot 2: Application home page of HealthHub app

This is the home page of the application showing its key functions, including making appointments, getting your medical reports, requesting for medication refills, and making payment amongst other functions.


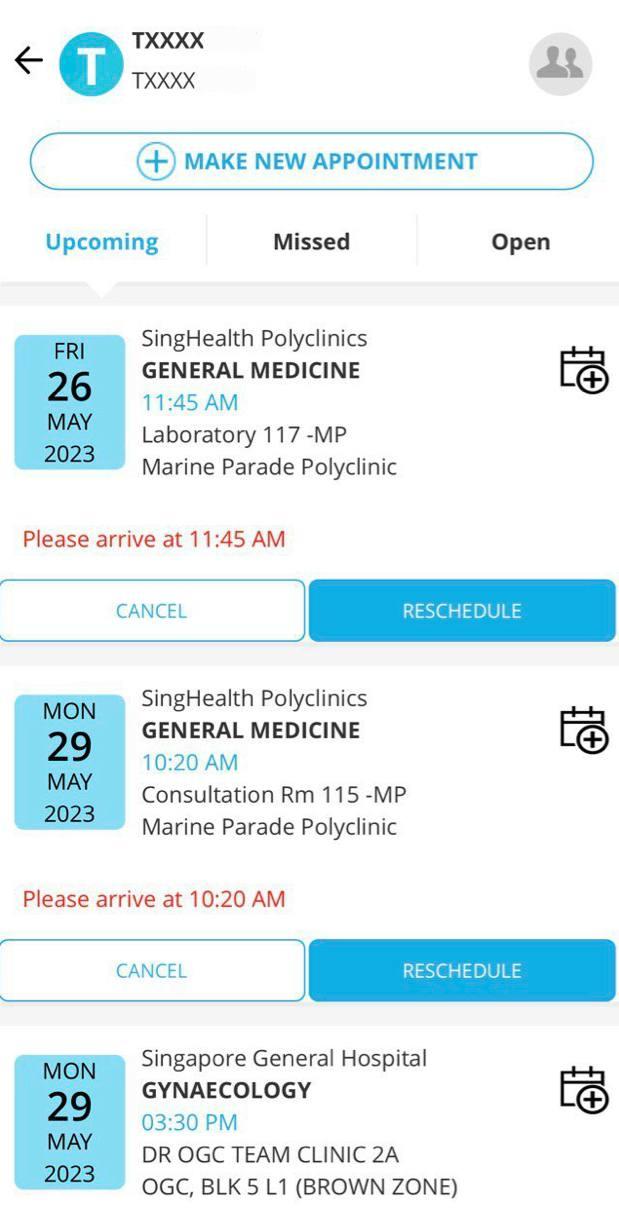


Screenshot 3: Page to manage appointments

On this page you can view all upcoming appointments, reschedule them as well as make new appointments.


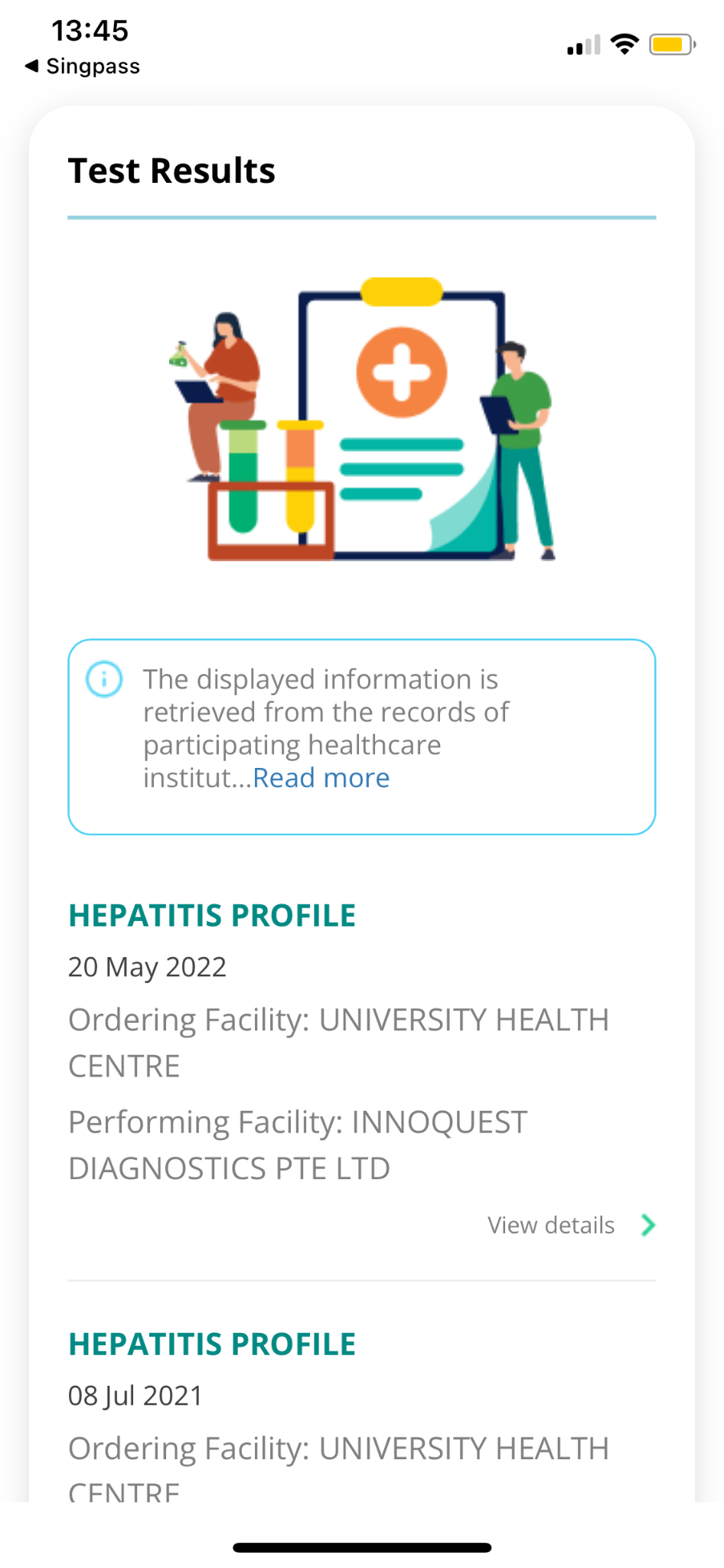


Screenshot 4: Page showing lab reports and test results

This page shows the lab reports and test results of all health screenings and tests that have been done.


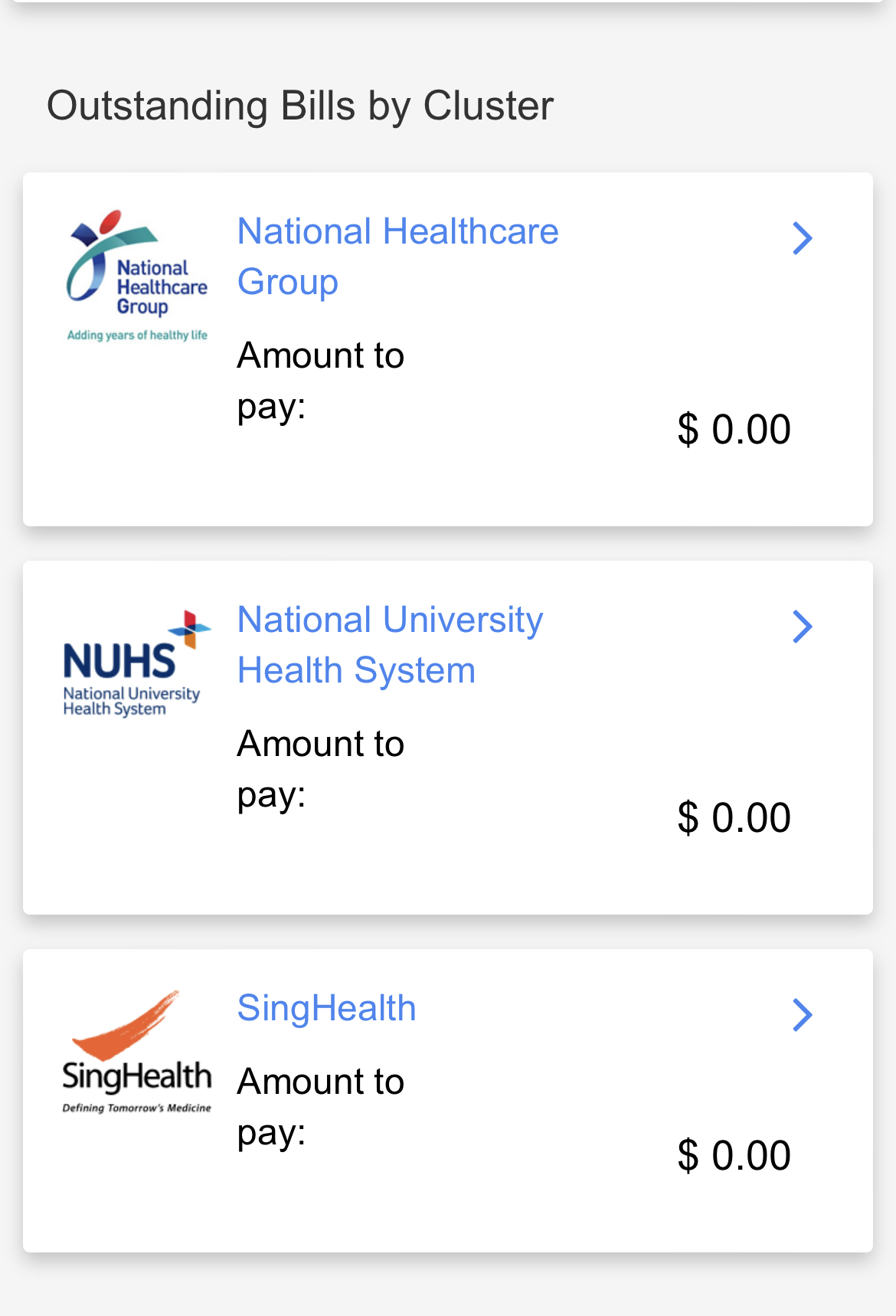


Screenshot 5: Page for (outstanding) payments

This page allows you to see any outstanding payments that you may have. You may also make payments through this page and reduce the time waiting at the clinics to make payment.

#

#


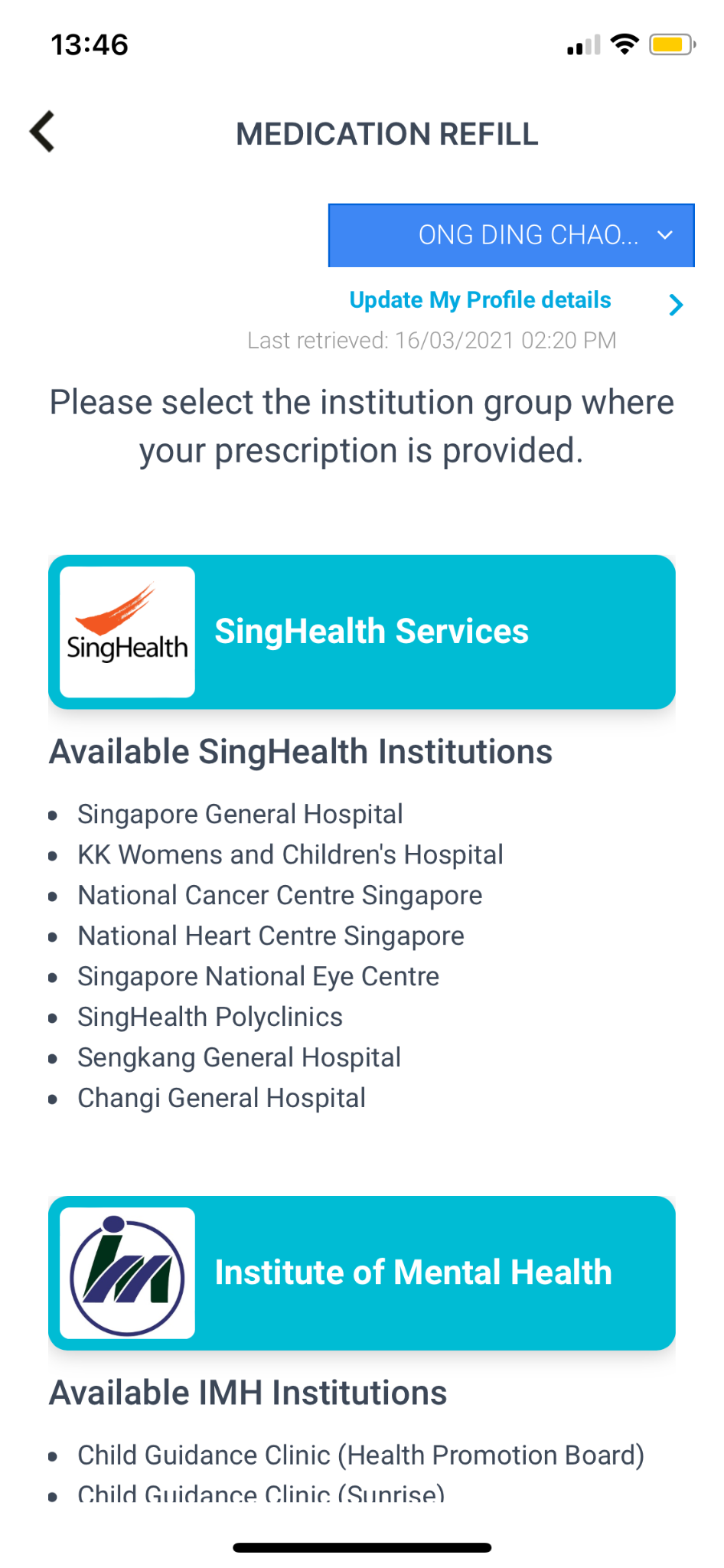

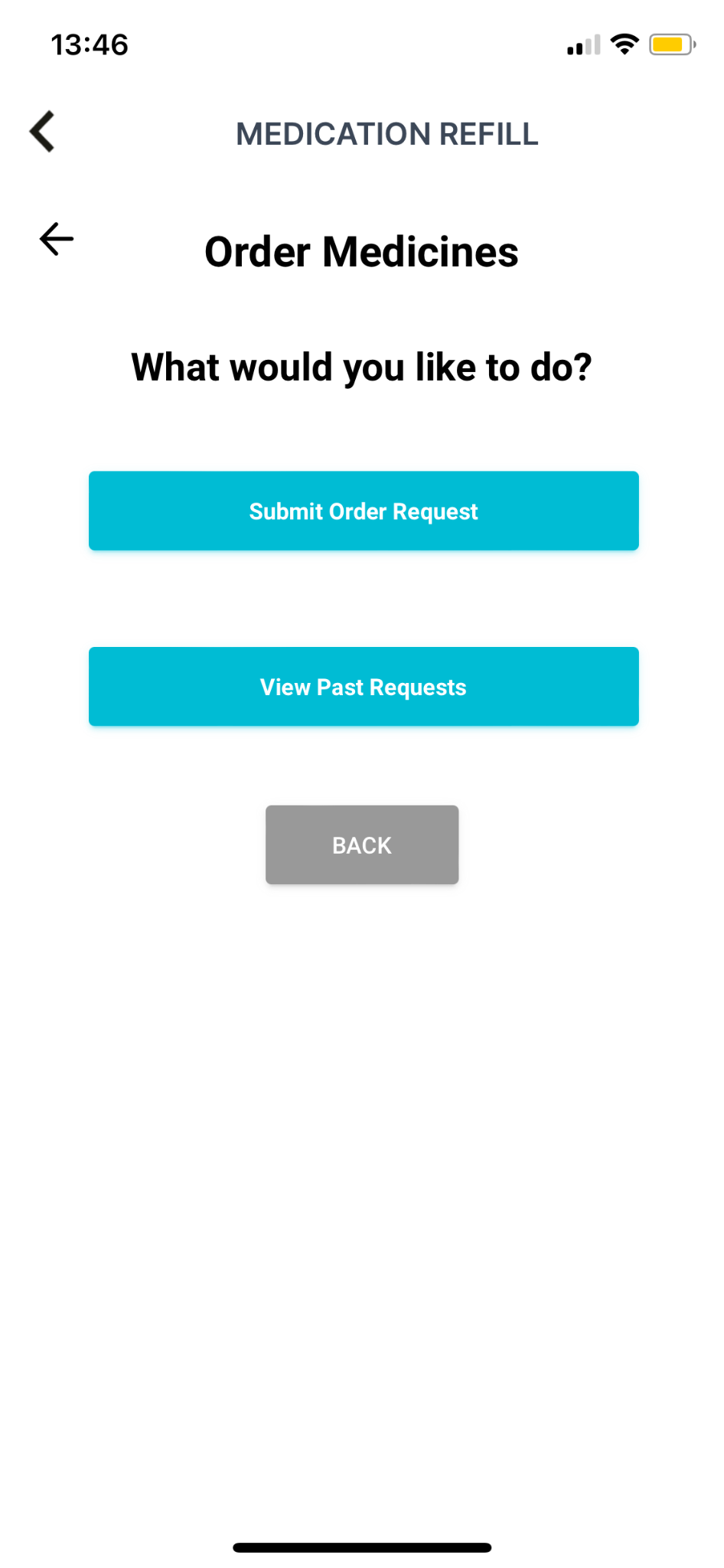


Screenshot 6: Page for medication request and refill

This page allows you to ask for a refill for certain medications (e.g. medication for chronic diseases such as diabetes, high blood pressure, etc.) that your doctor has previously prescribed.


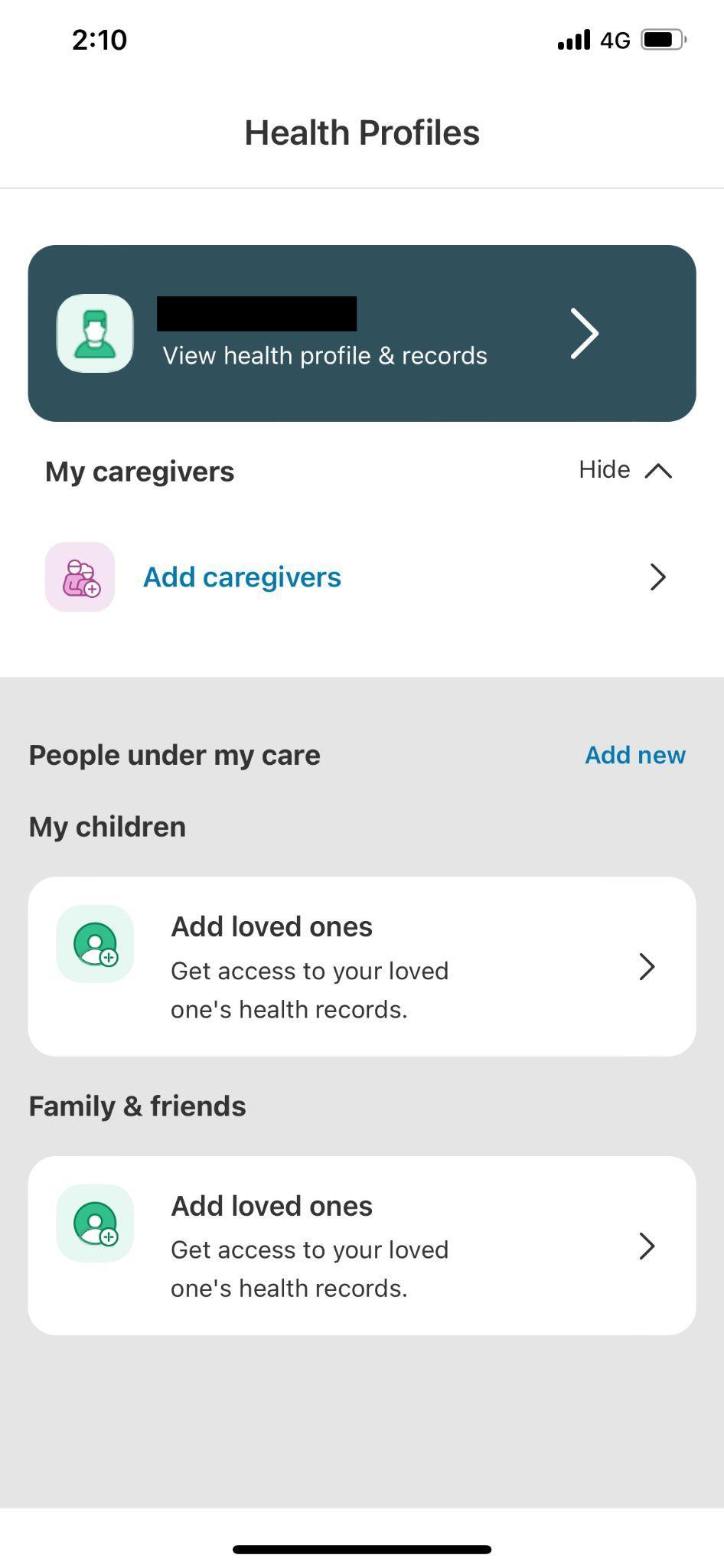


Screenshot 7: Page for health profiles

On this page, you can add the profiles of your caregivers to allow them to view your health records and appointments. You can add the profiles of people under your care (e.g. your children) to view their health records.


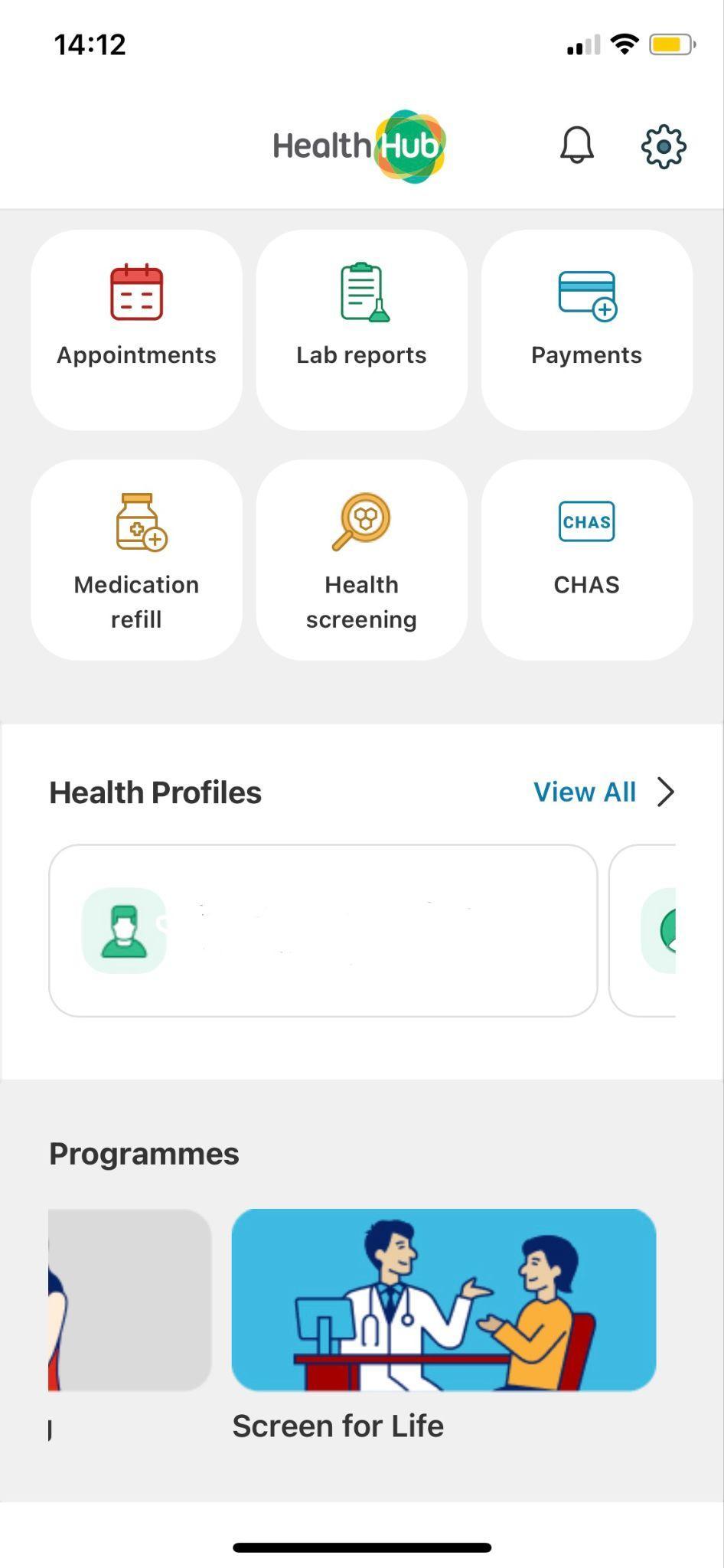

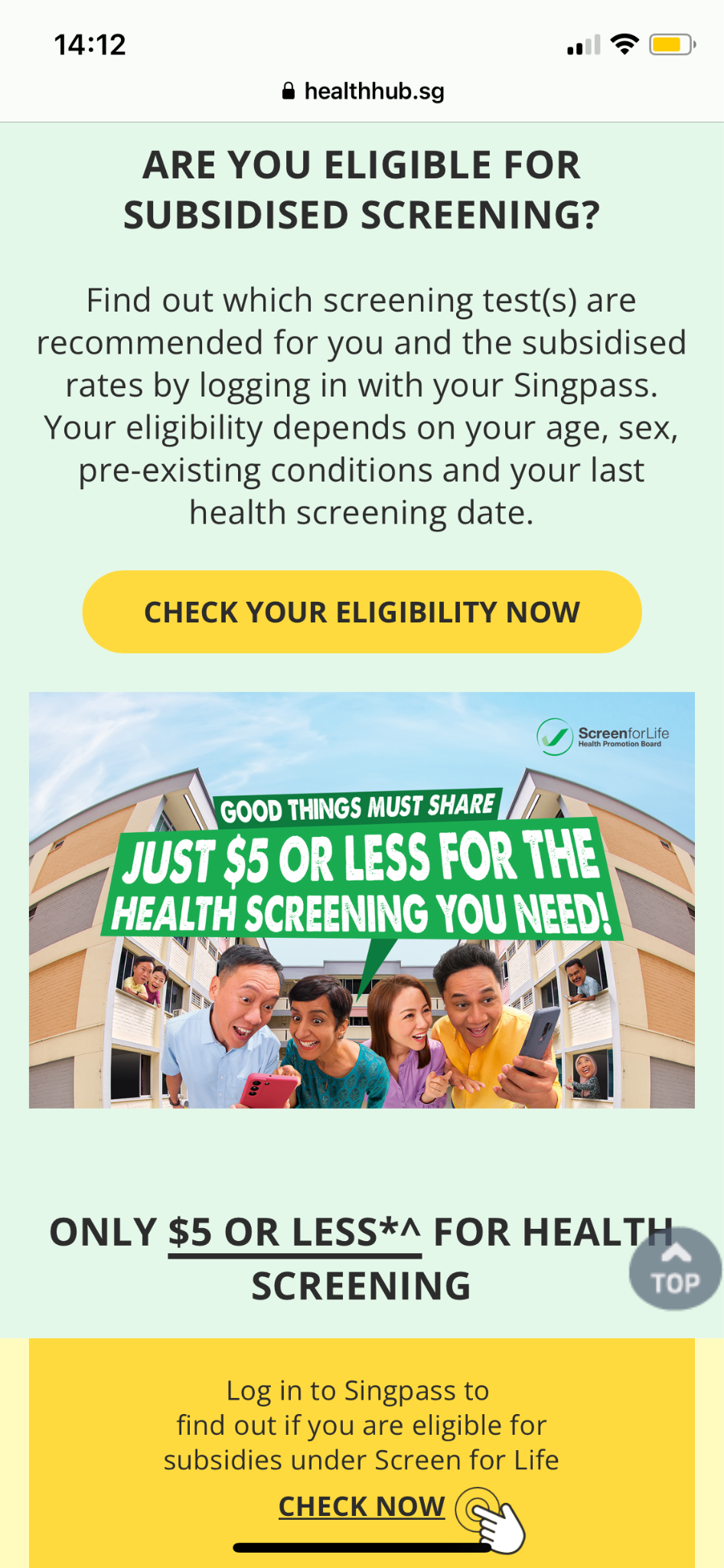

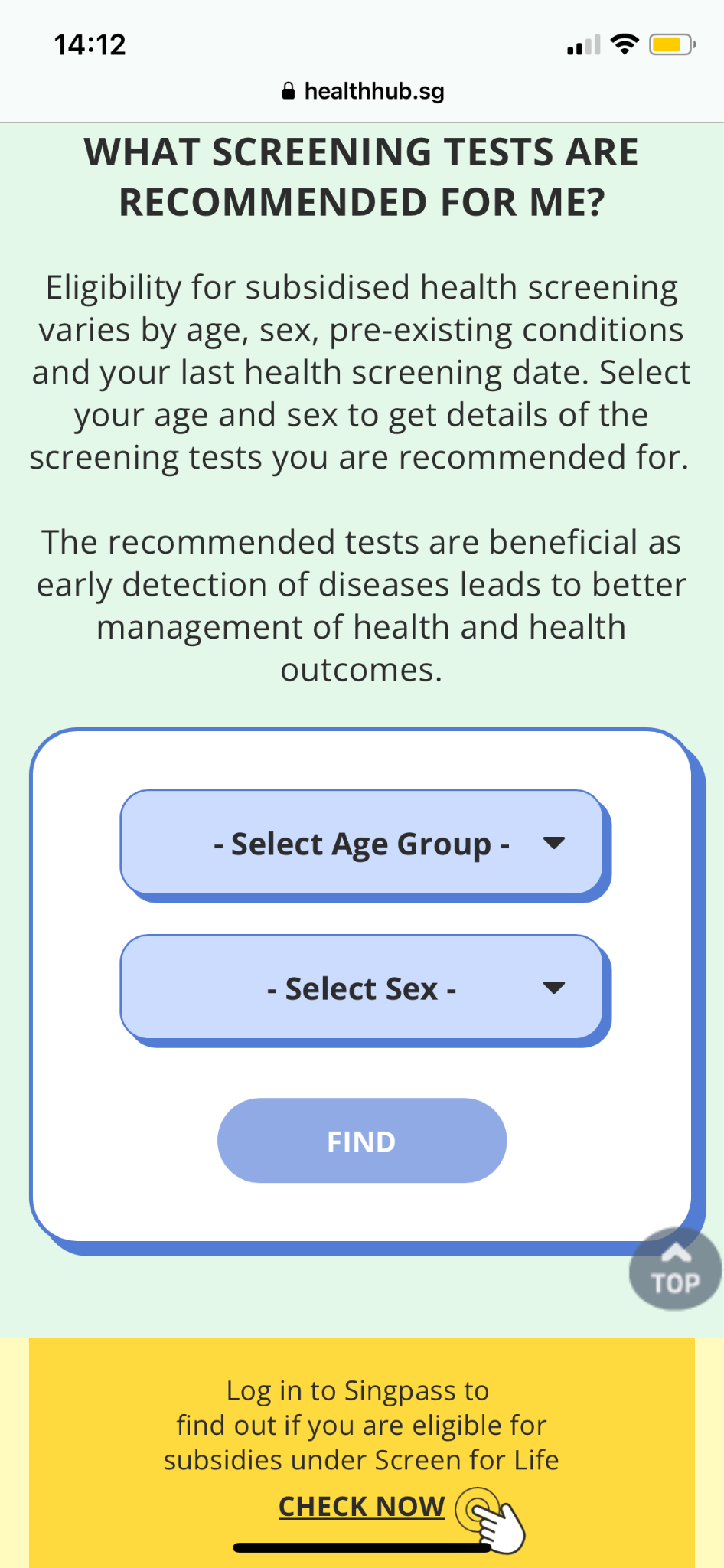


Screenshot 8: “Screen for Life” programme page

This page tells you what chronic disease and cancer screening tests you are eligible for, the cost of each test, and the nearest CHAS GP clinic to do the test.

#

#

#

#

#

# **Section D: Use of Technology in Health**

This section contains **7** questions to help us find out more about your views about the HealthHub app.

1. Have you used the HealthHub app before? Yes/No

**Instructions to participant:**

If you are a current user of the HealthHub app or have used it before, please answer the following questions based on your experience of using the app.

If you have not used the HealthHub app before, please answer the following questions based on what you have read in Section C.

1. I find the HealthHub app to be clear and understandable.

| Strongly Disagree | Disagree | Somewhat Disagree | Neutral | Somewhat Agree | Agree | Strongly Agree |
| --- | --- | --- | --- | --- | --- | --- |

1. I find the HealthHub app easy to use.

| Strongly Disagree | Disagree | Somewhat Disagree | Neutral | Somewhat Agree | Agree | Strongly Agree |
| --- | --- | --- | --- | --- | --- | --- |

1. I find it easy to get the HealthHub app to do what I want it to do.

| Strongly Disagree | Disagree | Somewhat Disagree | Neutral | Somewhat Agree | Agree | Strongly Agree |
| --- | --- | --- | --- | --- | --- | --- |

1. Using the HealthHub app supports important aspects of my healthcare.

| Strongly Disagree | Disagree | Somewhat Disagree | Neutral | Somewhat Agree | Agree | Strongly Agree |
| --- | --- | --- | --- | --- | --- | --- |

1. Using the HealthHub app increases my effectiveness in managing my healthcare.

| Strongly Disagree | Disagree | Somewhat Disagree | Neutral | Somewhat Agree | Agree | Strongly Agree |
| --- | --- | --- | --- | --- | --- | --- |

1. I find the HealthHub app to be useful in managing my healthcare.

| Strongly Disagree | Disagree | Somewhat Disagree | Neutral | Somewhat Agree | Agree | Strongly Agree |
| --- | --- | --- | --- | --- | --- | --- |
